# Single-cell hdWGCNA identifies checkpoint inhibitor pneumonitis-linked CD4 + T-cell subsets driving immunotherapy response and prognosis in lung cancer

**DOI:** 10.1007/s00432-026-06522-9

**Published:** 2026-07-10

**Authors:** Qiuming Chen, Shaocong Mo, Jun Cheng, Linhai Zhu

**Affiliations:** 1 Department of Thoracic Surgery, The First Affiliated Hospital, Zhejiang University School of Medicine, Hangzhou, Zhejiang China; 2 Department of Digestive Diseases, Huashan Hospital, Fudan University, Shanghai, China

**Keywords:** Checkpoint inhibitor-related pneumonitis, Lung cancer, CD4 + T cells, Single-cell RNA-seq, hdWGCNA

## Abstract

**Background:**

Immune checkpoint inhibitors (ICIs) have achieved promising efficacy in lung cancer patients, yet checkpoint inhibitor-related pneumonitis (CIP) remains a severe and incompletely understood complication. The pathogenesis of CIP and its impact on immunotherapy outcomes remain elusive, especially the role of specific immune cell subsets in mediating these processes.

**Methods:**

Single-cell RNA sequencing (scRNA-seq) data from CIP patients were analyzed to delineate the distinct immune microenvironment landscape. High-dimensional weighted gene co-expression network analysis (hdWGCNA) was performed to identify CIP-related CD4 + T cell gene modules. Key results were validated in an independent lung adenocarcinoma (LUAD) cohort, and a prognostic signature was constructed. Immunofluorescence staining was applied to verify the existence of the identified T-cell subsets in CIP-lung tissues.

**Results:**

Our analysis uncovered prominent CD4 + T cell infiltration in the CIP immune microenvironment. hdWGCNA identified key CIP-associated CD4 + T cell genes enriched in pathways including viral response, cytokine production and T cell differentiation. Validation in the LUAD cohort verified robust associations between these CD4 + T cell subsets and both patient prognosis and immunotherapy response. The constructed prognostic signature exhibited favorable predictive performance, with BIRC3 identified as the leading risk gene. Immunofluorescence staining confirmed the spatial localization of BIRC3 + CD4+ T cells in CIP patients.

**Conclusion:**

This study identifies critical CD4 + T cell subpopulations mediating CIP pathogenesis and establishes a robust gene signature for predicting survival and ICI response in LUAD patients. Our findings provide mechanistic insights into CIP progression and offer potential therapeutic targets for the clinical management of this life-threatening immunotherapy complication.

**Supplementary Information:**

The online version contains supplementary material available at https://doi.org/10.1007/s00432-026-06522-9.

## Introduction

Over the past few decades, lung cancer has become the most prevalent malignancy and the leading cause of cancer-related mortality worldwide (Siegel et al. 2022), posing a severe threat to human health (Sung et al. 2021). Accordingly, current lung cancer research has focused primarily on developing novel preventive and therapeutic strategies (Allemani et al. 2018).

Immune checkpoint inhibitors (ICIs) represent a novel class of therapeutic agents that have yielded substantial clinical benefits across multiple cancer types (Lee et al. 2022; Topalian et al. 2012). ICIs target immune checkpoint receptors on immune cells and block their inhibitory signals, enhancing the ability of immune cells to recognize and clear tumor cells, thus accelerating tumor ablation (He et al. 2021; Shiravand et al. 2022). Although ICIs are well tolerated in patients with non-small cell lung cancer (NSCLC) and have improved survival outcomes (Suresh et al. 2018), unprecedented immune-related adverse events (irAEs) have been observed in many organs, including the skin, lung and thyroid (Aldarouish and Wang 2016). Checkpoint inhibitor-related pneumonitis (CIP) is one of the most common life-threatening irAEs, which may occur at any stage during ICI therapy (Frost et al. 2023; Tiu et al. 2022). However, the underlying mechanisms of CIP remain incompletely elucidated and warrant further exploration.

The occurrence of CIP may be related to enhanced cross-reactivity of T cells against antigens shared by tumor and normal tissues. Within T lymphocytes, the proportion of central memory T cells (Tcm) increases, while the expression of CTLA-4 and PD-1 decreases, exerting negative regulatory effects on CD8 + T cells, conventional T cells, and proinflammatory responses in macrophages (Zhai et al. 2020). An increase in activated pulmonary interstitial lymphocytes and a decrease in anti-inflammatory regulatory T cells may lead to T-cell dysfunction. Activated T lymphocytes not only mediate tumor cell killing but also damage alveolar epithelial cells, thereby contributing to CIP (André et al. 2020).

CD4 + T cells are essential components of the immune system and play a pivotal role in tumor immunity (Kennedy and Celis 2008). They secrete IL-2 as a co-stimulatory signal to activate CD8 + T cells and initiate cytotoxic anti-tumor immune responses (Speiser et al. 2023) Studies have shown that patients with ICI-induced arthritis present reduced CD8 + T cell counts, whereas those with pneumonitis exhibit elevated CD4 + T cell levels. Patients with thyroiditis show increased abundance of CD4 + Th17 cells compared with those without irAEs, suggesting a closer association of CD4 + T cells with pneumonitis than CD8 + T cells.

Recently, the rapid advancement of scRNA-seq has provided a powerful tool for dissecting the heterogeneity of tumor-infiltrating immune cells (Ding et al. 2025; Zhang and Zhang 2019). For instance, Zhang et al. (2025) integrated scRNA-seq with machine learning to characterize immunogenic cell death‑related features in LUAD, and Cheng et al. (2026) employed an integrative single‑cell and machine learning framework to construct a fibroblast‑related risk signature in LUAD. However, no studies have yet used the unique immunological perspective provided by CIP to explore LUAD.

In this study, we performed high-dimensional weighted gene co-expression network analysis (hdWGCNA) to identify genes associated with specific CD4 + T cell subpopulations in lung cancer patients with CIP and explored the underlying biological pathways using Metascape. We further identified CIP-related CD4 + T cell subtypes in LUAD and investigated their associations with immunotherapy response and prognosis. Furthermore, we constructed a prognostic model based on 11 CIP–CD4 + T cell-related genes, stratifying patients into high- and low-risk groups, which was validated across multiple cohorts. Immune cell infiltration and immune checkpoint expression were also analyzed. Additionally, TIDE scores were calculated to evaluate immune evasion potential. Our findings provide novel insights into the mechanisms underlying CIP development and identify potential therapeutic targets to improve the prognosis of lung cancer patients.

## Methods

### Data extraction and collation

ScRNA-seq data from 11 cancer patients with CIP and six demographically matched control patients were obtained from a previous study by Amelie et al. (2022) and downloaded from the website of the VIB-KU Leuven Center for Cancer Biology (detailed clinical characteristics are provided in Supplementary Table S1). In addition, mRNA expression profiles, clinical characteristics, and mutation annotation data of 524 lung adenocarcinomas (LUAD) and 58 normal samples were collected from The Cancer Genome Atlas (TCGA) database. The expression matrix and corresponding clinical information of 443 tumor samples from the GSE68465 cohort were used for external validation.

This study was performed in accordance with the Declaration of Helsinki. Approval was granted by the Ethics Committee of The First Affiliated Hospital, Zhejiang University School of Medicine (2024-06-27/No. 2024-0095). Written patients informed consent was waived by the Institutional Review Board because of the retrospective study.

### Single-cell RNA-seq analysis of CIP samples

The Seurat object was initialized and transcriptomic data were processed using the R package “Seurat”. Cells with > 2500 or < 200 detected genes and mitochondrial gene ratio > 20% were excluded, to remove damaged or dying cells while retaining the population of viable cells (Franken et al. 2022). After normalization, data integration was performed using the RunHarmony function from the R package “Harmony”, with “orig.ident” as a covariate (theta set at 2, sigma set at 0.1, and lambda set at 1). Principal component analysis (PCA) was conducted based on highly variable genes, and cell types were annotated using canonical marker genes. CD4 + T cell clusters were further extracted for subclustering analysis, and the proportions of cell subsets in CIP and control groups were calculated. Pseudotime trajectory analysis of CD4 + T cells was performed using Monocle2, and cell–cell communication analysis was conducted using CellChat.

### High dimensional weighted gene co-expression network analysis (hdWGCNA)

The hdWGCNA was used to identify genes significantly correlated with CD4 + T cells in CIP samples. Briefly, a gene expression correlation matrix of CD4 + T cells was constructed based on scRNA-seq data to build a weighted co-expression network. We input the genes expressed in at least 5% of the cells. We used the MetacellsByGroups function via the R package “hdWGCNA” to construct metacells by grouping cells from the same sample and cell type, using Harmony-reduced PCA space with K-nearest neighbors parameters (k set at 20, max_shared set at 10, min_cells set at 100). The soft threshold power was determined using a scale-free topology criterion. Gene modules were detected using the dynamic tree-cutting algorithm, and similar modules were merged. Module–trait relationships were analyzed to identify CIP-associated modules. The GetHubGenes function in the R package “hdWGCNA” was used to identify hub genes in the key module, with the parameter n_hubs set to 50 to select the top 50 hub genes. The top 50 hub genes in the key module were identified according to intramodular connectivity and defined as CIP-related CD4 + T cell signature genes.

### Functional analysis and network of CIP-CD4 + T cell genes

The interaction network of the 50 hub genes was constructed using the GeneMANIA database. Functional and pathway enrichment analyses were performed using Metascape with a cutoff of *P* < 0.01 and gene count ≥ 3. Differentially expressed CIP-related CD4 + T cell genes between normal lung and LUAD tissues were screened using the R package “limma”(Ritchie et al. 2015). Somatic mutation profiles of the TCGA-LUAD cohort were analyzed and visualized using the “maftools” package (Mayakonda et al. 2018).

### Identification of CIP-related CD4 + T cell molecular subtypes

Unsupervised consensus clustering was performed using the R package “ConsensusClusterPlus” to identify molecular subtypes based on the expression of CIP-related CD4 + T cell genes in the TCGA-LUAD cohort (Wilkerson and Hayes 2010). The clustering analysis was repeated 1000 times to ensure stability.

### Impact of CIP-CD4 + T cell subtypes on the immunotherapeutic response

The response of CIP-CD4 + T cell subgroups to immunotherapy was predicted using the TIDE database (http://tide.dfci.harvard.edu), based on LUAD transcriptome data. In addition, the immune prognostic scores (IPS) of patients with LUAD in response to ICIs were retrieved from the TCIA (https://tcia.at/) database to determine the predictive potential of CIP-CD4 + T cell subtypes. Gene set enrichment analysis (GSEA) was performed to explore underlying biological mechanisms (Subramanian et al. 2005).

### Construction and validation of a prognostic signature

Prognosis-related genes were initially screened using univariate Cox regression analysis. Least absolute shrinkage and selection operator (LASSO) regression was applied to filter genes and avoid overfitting. The R package “glmnet” parameter was used to perform LASSO analysis, with family set to “cox” in order to fit a Cox proportional hazards model. The alpha parameter was set to 1 to apply L1 regularization (i.e., the LASSO penalty term) for penalizing the regression coefficients. Ten-fold cross-validation (nfolds = 10) was adopted to evaluate model stability and select the optimal regularization parameter λ. A risk score formula was established, and patients were stratified into high- and low-risk groups using the median risk score as the cutoff. Survival analysis was performed using the Kaplan–Meier method with the log-rank test.

### Immune infiltration analysis

Immune, Stromal, and ESTIMATE scores were ascertained using the ESTIMATE algorithm (Yoshihara et al. 2013). The proportions of 22 tumor-infiltrating immune cell types were quantified using the CIBERSORT algorithm (Newman et al. 2015). Violin plots were used to visualize immune infiltration differences between risk groups. The correlation between risk score and immune checkpoint gene expression was also analyzed.

### Immunofluorescence staining for lung cancer tissue sections

FFPE lung tissue sections were deparaffinized, rehydrated, and subjected to antigen retrieval in EDTA buffer (pH 9.0). After blocking, sections were incubated with anti-BIRC3 and anti-CD4 primary antibodies overnight at 4 °C. TSA-based dual-color immunofluorescence was performed using Opal 690 and Opal 570 reagents. Nuclei were counterstained with DAPI. Images were acquired using a fluorescence microscope with appropriate filter sets.

### Statistical analysis

All statistical analyses were performed using R software (version 4.2.3). A *P* value < 0.05 was considered statistically significant.

## Results

### Single-cell analysis reveals the immune landscape of CIP

**Fig. 1:**
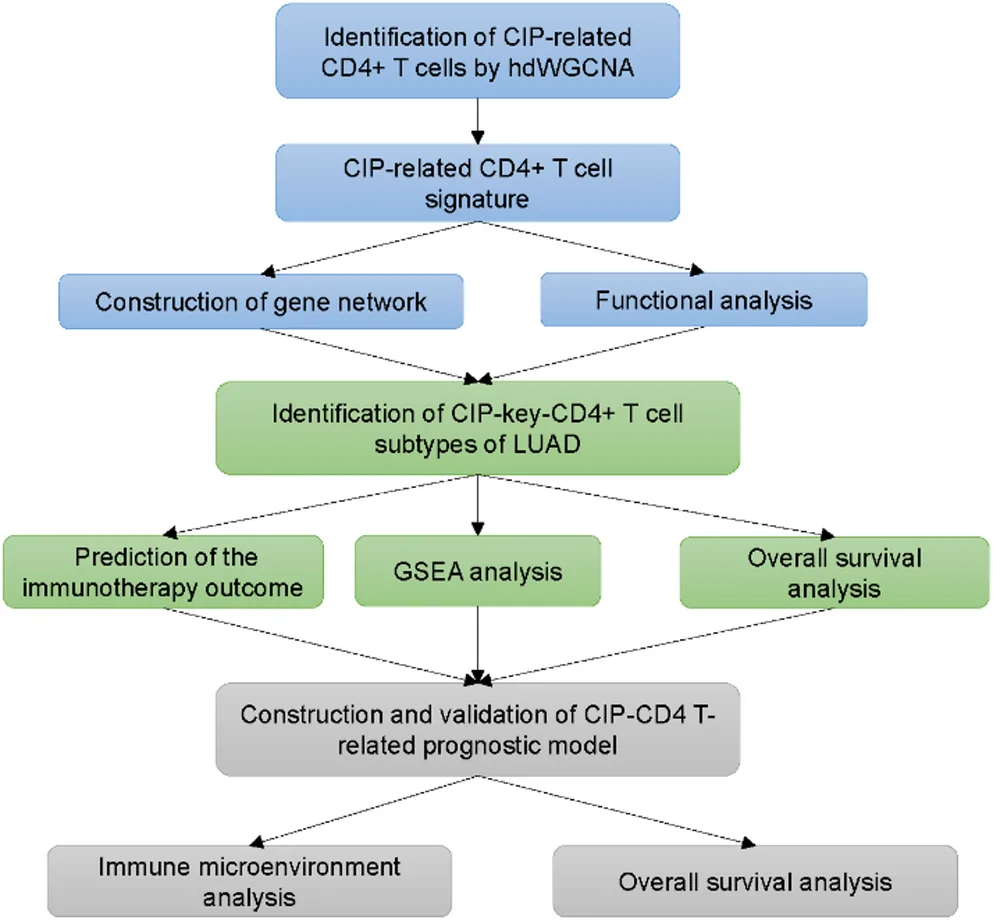
Workflow of the study

**Fig. 2:**
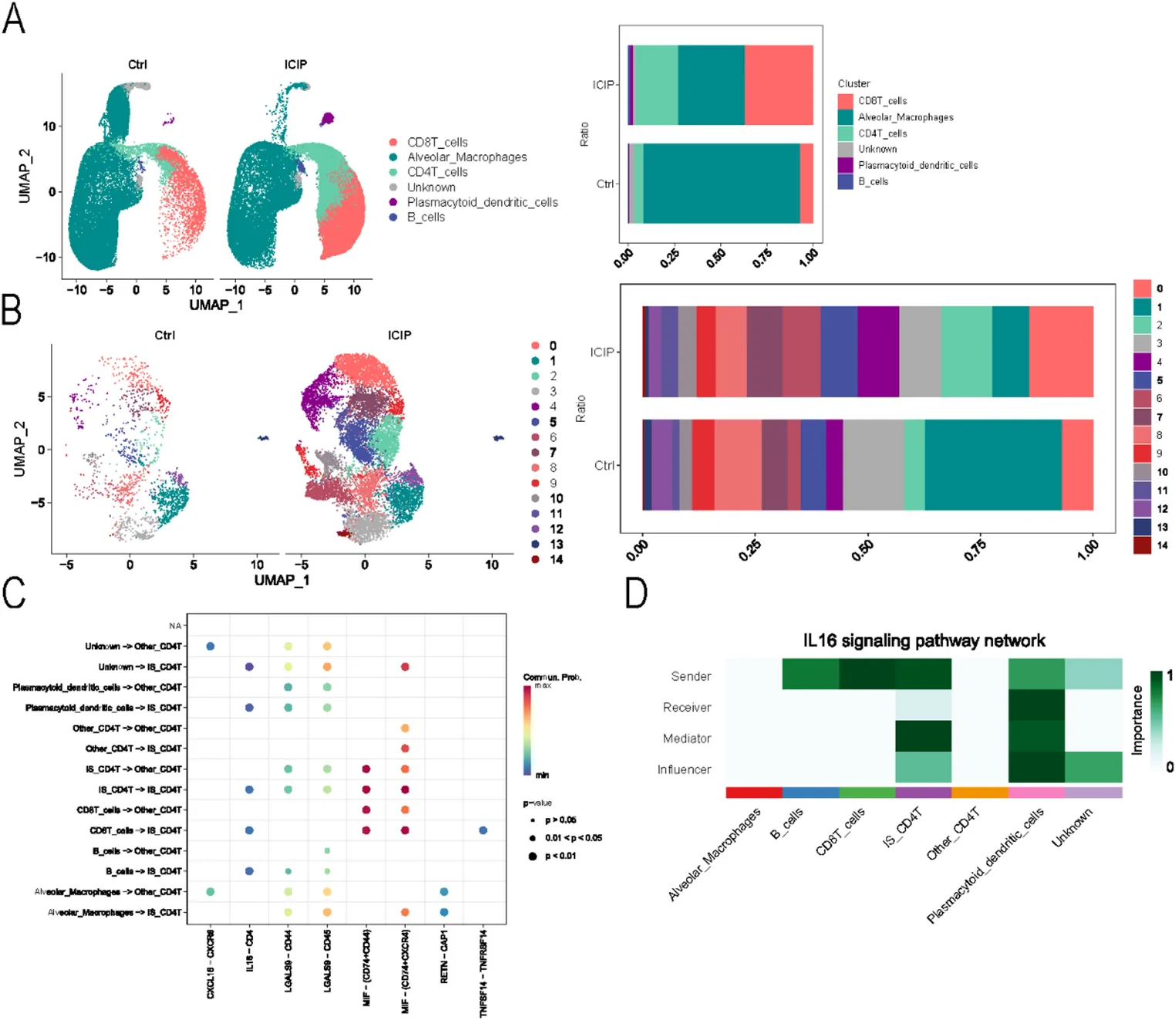
ScRNA-seq analysis of CD4 + T cells in CIP samples.**A** Clustering and the proportion of different cell subpopulations in the Control and CIP groups. **B** Clustering and the proportion of different CD4 + T cell subpopulations in the Control group and CIP group. **C** Cellchat analysis of all cell types. **D** Heatmap showing the roles of the different CD4 + T cell clusters in the IL-16 signaling network

The flowchart of this study was shown in Fig. 1. A total of 88,316 qualified cells from 11 CIP and 6 control samples were included. Five major cell types were identified, including plasmacytoid dendritic cells, alveolar macrophages, CD4 + T cells, CD8 + T cells, and B cells (Figs. 2A and S1A–D). The CIP group exhibited significantly higher infiltration of CD4 + T cells. Subclustering of CD4 + T cells identified 15 subsets, among which Cluster 0 was remarkably enriched in CIP (Fig. 2B). Cell–cell communication analysis showed that CD4 + T cells were involved in multiple signaling pathways, including CXCL16, IL-16, LGALS9, MIF, RETN, and TNFSF14. Notably, Cluster 0 acted as both a sender and mediator in the IL-16 pathway (Fig. 2C and D). These results highlight the unique distribution and functional role of CD4 + T cells in the CIP microenvironment.

### HdWGCNA identifies key CD4 + T cell gene modules in CIP

**Fig. 3:**
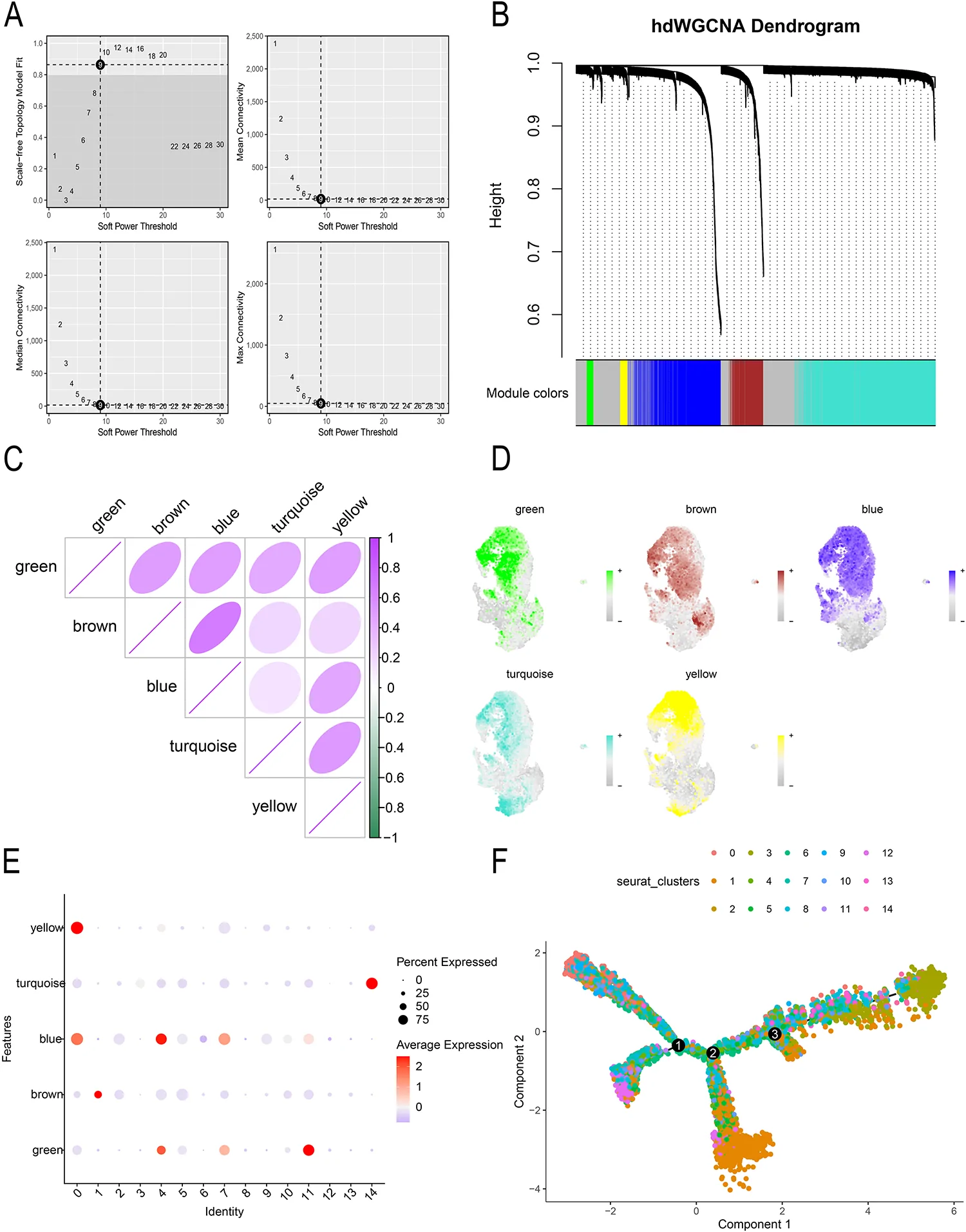
The yellow module of CD4 + T cells plays a key role in CIP.**A** Selection of the optimum soft threshold. **B** A clustering tree and co-expression network were built based on the optimal soft threshold of “9” by dividing the genes into five modules. **C** Correlation between the 5 modules. **D** CD4 + T cell UMAP colored by ME. **E** Module activities in the 15 CD4 + T cell clusters. **F** Pseudotime distribution of the 15 CD4 + T cells clusters

A scale-free co-expression network was constructed with a soft power of 9 (Fig. 3A). Five gene modules were identified (Figs. 3B and S2A), and the correlation is shown in Fig. 3C. Module–trait correlation analysis revealed that the yellow module was significantly activated in Cluster 0 (Fig. 3D, E), suggesting a close association with CIP. Pseudotime analysis showed that Cluster 0 was mainly distributed in the early developmental branches (Figs. 3F and S2B–D), indicating a relatively immature state.

### Functional characteristics of CIP-related CD4 + T cell genes

**Fig. 4:**
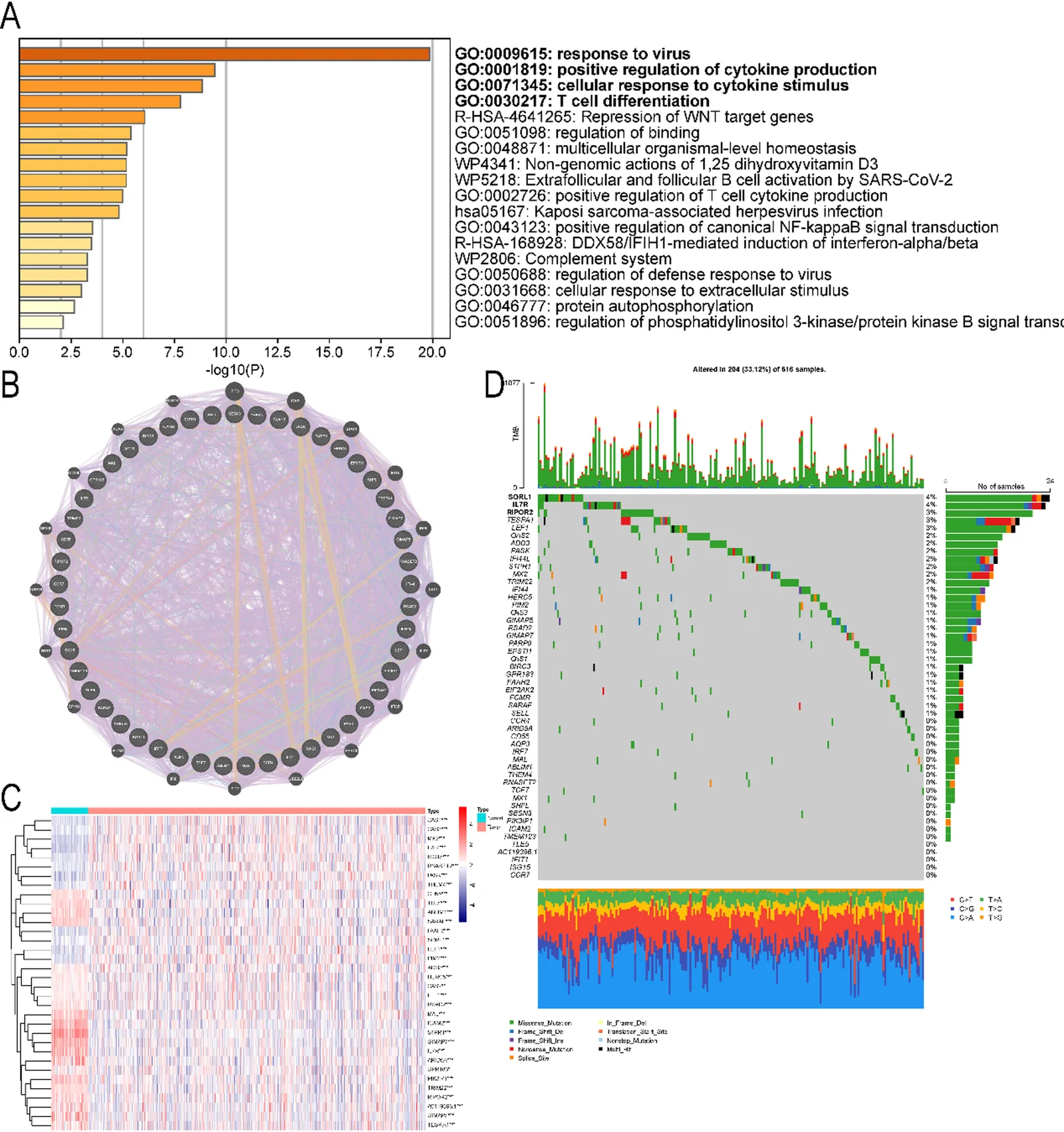
Functional analysis of CIP-key-CD4 + T cell genes. **A** Bar chart illustrating the enriched functions in CIP-CD4 + T cell genes. **B** Gene network of CIP-CD4 + T cell genes. **C** Heatmap showing the CIP-CD4 + T cell genes in LUAD and normal tissues of TCGA-LUAD cohort. **D** Waterfall plot showing the mutation frequencies of CIP-CD4 + T cell genes in the TCGA-LUAD cohort

The interaction network of the top 50 hub genes was constructed using GeneMANIA (Fig. 4B). Enrichment analysis showed that these genes were mainly involved in T cell differentiation, response to viral infection, positive regulation of cytokine production, and cellular response to cytokine stimuli (Fig. 4A). Differential expression analysis demonstrated distinct expression patterns of these genes between normal and LUAD tissues (Fig. 4C). Mutation analysis revealed relatively high mutation frequencies of SORL1, IL7R, and RIPOR2 in LUAD (Fig. 4D).

### Identification of CIP-key-CD4 + T cell subtypes of LUAD

**Fig. 5:**
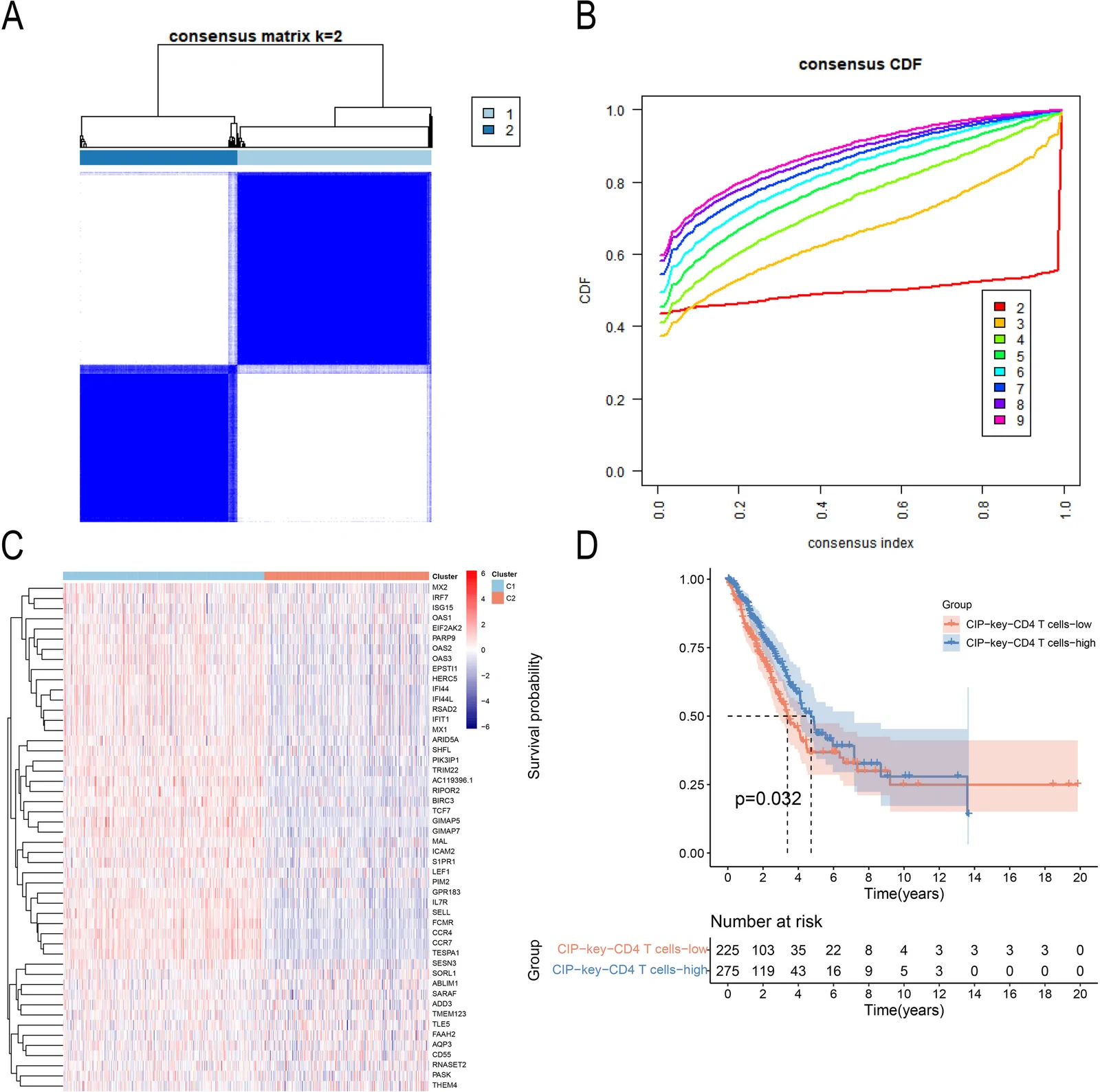
Identification of CIP-CD4 + T cell subtypes of LUAD. **A** The CDF curve of the clustering result. **B** Consensus score matrix of all samples at k = 2. **C** Heatmap of CIP-CD4 + T cell genes in Cluster1 and Cluster2. **D** Kaplan-Meier survival curves of CIP-CD4 + T cell-based clusters in the TCGA-LUAD cohort

After performing univariate Cox regression analysis on the 50 hub genes, we identified 17 genes associated with the prognosis of LUAD: AQP3, BIRC3, CCR4, CCR7, EPSTI1, FCMR, GIMAP5, GIMAP7, ISG15, MAL, OAS1, OAS3, PIK3IP1, PIM2, RIPOR2, TESPA1, and TRIM22. Patients with LUAD were categorized based on the expression levels of 17 prognostic genes. Consensus clustering stratified LUAD patients into two clusters (k = 2) based on the expression of CIP-related CD4 + T cell genes (Fig. 5A, B). The expression patterns of 50 CIP-CD4 + T cell genes in the two clusters were then evaluated. As shown in Fig. 5C, most genes were downregulated in cluster 2 (C2) and strongly expressed in cluster 1 (C1) and were designated as CIP-CD4 + T cells^low^ and CIP-CD4 + T cells^high^. Kaplan–Meier survival analysis showed that patients in the CIP-CD4 + T cellshigh group had significantly longer overall survival (Fig. 5D). The findings elucidated the importance of CIP-CD4 + T cell gene expression for molecular subtyping in LUAD patients.

### CD4 + T cell subtypes predict immunotherapy response

**Fig. 6:**
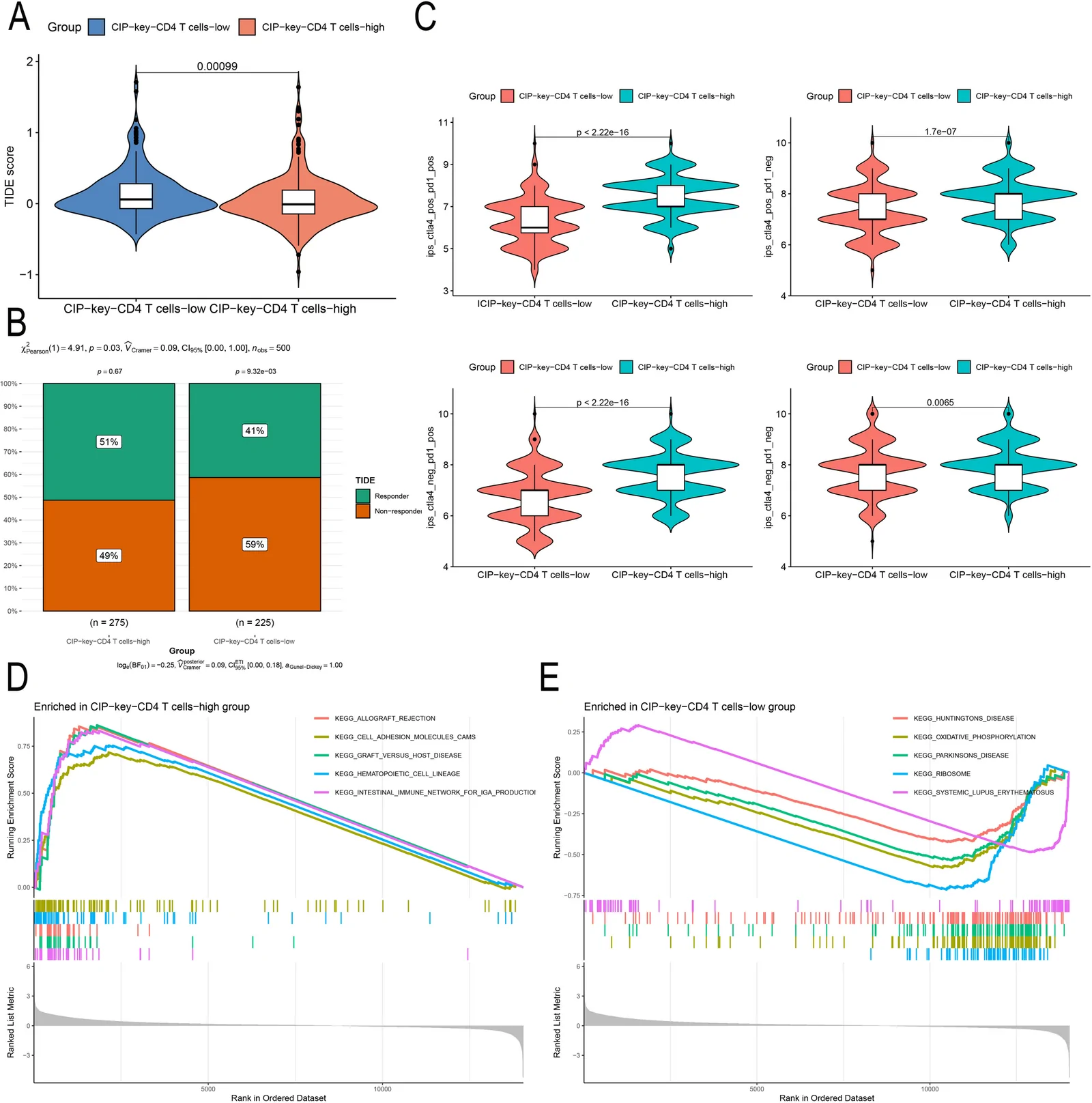
Immunotherapy outcomes in CIP-CD4 + T cell-based patient clusters. **A** TIDE scores of the CIP-CD4 + T cells^low^ and CIP-CD4 + T cells^high^ patients from the TCGA-LUAD cohort **B** Percentage of patients in the CIP-CD4 + T cells^low^ and CIP-CD4 + T cells^high^ groups that can likely respond to immunotherapy. **C** IPS scores in the CIP-CD4 + T cells^low^ and CIP-CD4 + T cells^high^ LUAD patients. **D** GSEA results of the CIP-CD4 + T cells^high^ group. (E) GSEA results of CIP-CD4 + T cells^low^ group

Immunotherapy outcomes in the CIP-CD4 + T cells^low^ and CIP-CD4 + T cells^high^ groups were analyzed using the TIDE algorithm. TIDE scores were significantly higher in the CIP-CD4 + T cell^slow^ group, indicating stronger immune evasion and poorer ICI response (Fig. 6A and B). The IPS analysis confirmed better immunotherapy outcomes in the CIP-CD4 + T cells^high^ group (Fig. 6C). GSEA showed that the high-expression group was enriched in allograft rejection and cell adhesion molecules pathways, while the low-expression group was enriched in Huntington’s disease and oxidative phosphorylation (Fig. 6D, E).

### Construction and validation of CIP-CD4T-related prognostic model

**Fig. 7:**
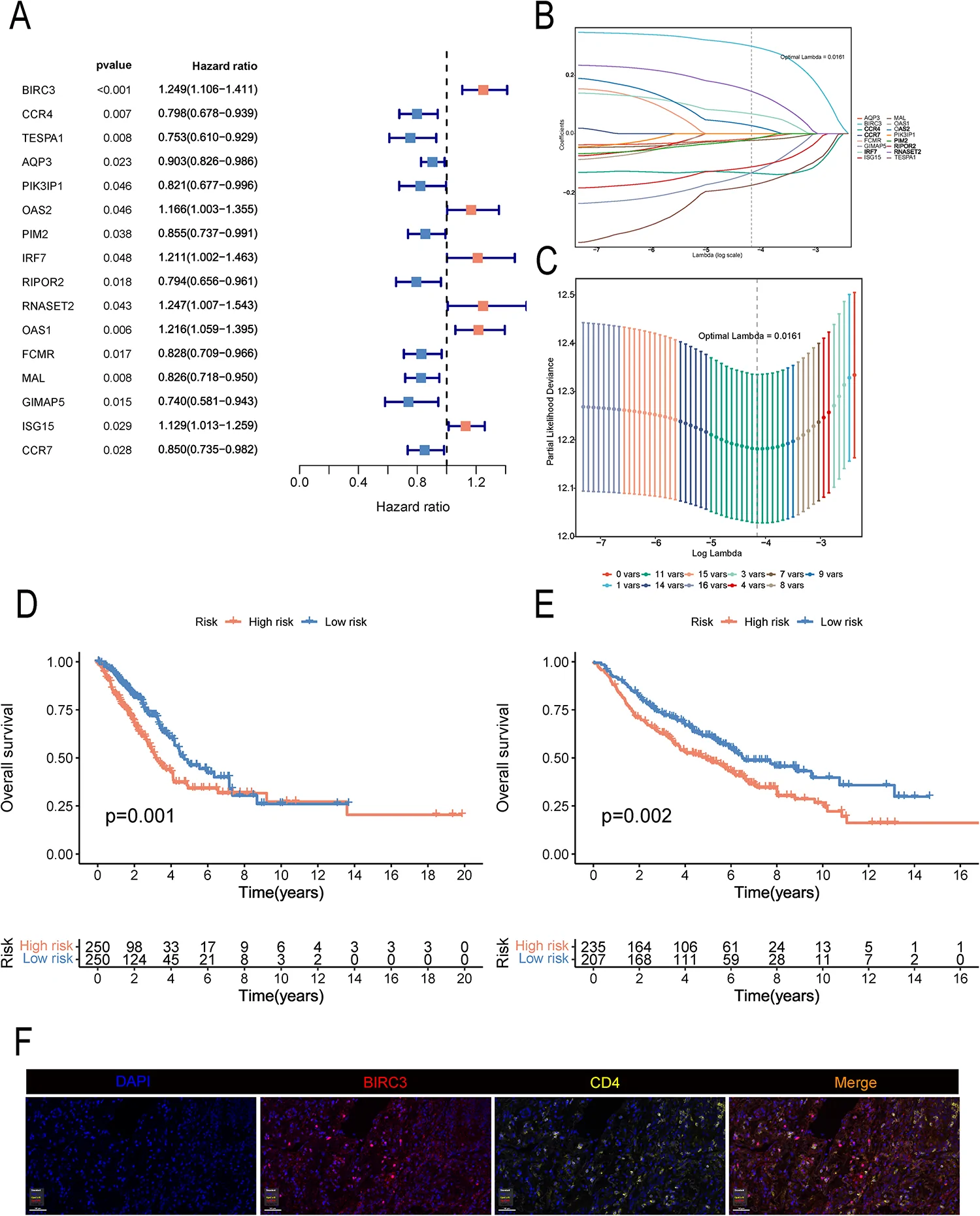
Development and validation of the risk-score model using CIP-CD4 + T cell genes. **A** Univariate regression analysis results reveal the significant correlation between CIP-CD4 + T cell genes and OS in LUAD patients from the TCGA cohort. **B** LASSO coefficient profiles for the 11 prognostic CIP-CD4 + T cell genes. **C** Visualization of the coefficient profiles with a logarithmic lambda sequence. **D** Kaplan-Meier curves illustrate OS differences between low-risk and high-risk groups in the TCGA-LUAD cohort. **E** Kaplan-Meier curves display OS distinctions between low-risk and high-risk groups in the GSE68465 dataset. **F** The immunofluorescence of the most significant risk genes BIRC3 and CD4 in lung tissues of CIP patients

After removing batch effects from the TCGA-LUAD and GSE68465 datasets, the univariate Cox regression analysis was performed to identify CIP-CD4⁺ T-cell genes significantly associated with LUAD patient survival, resulting in 16 prognostic genes: BIRC3, CCR4, TESPA1, AQP3, PIK3IP1, OAS2, PIM2, IRF7, RIPOR2, RNASET2, OAS1, FCMR, MAL, GIMAP5, ISG15, and CCR7 (Fig. 7A). LASSO regression was then performed to eliminate overfitting, with lambda = 0.0161 selected as the optimal value based on cross-validation. A total of 11 genes (BIRC3, CCR4, TESPA1, AQP3, OAS2, PIM2, IRF7, RIPOR2, RNASET2, MAL, and GIMAP5) were selected to construct the prognostic model (Figs. 7B, C and S3A, B). The corresponding flowchart (Fig. S4) provides an overview of this stepwise gene selection pipeline.

The risk score was calculated using the following formula: Risk score = (ExpBIRC3 × 0.3002) + (ExpCCR4 × -0.1352) + (ExpTESPA1 × -0.1771) + (ExpAQP3 × -0.0165) + (ExpOAS2 × 0.0276) + (ExpPIM2 × -0.0186) + (ExpIRF7 × 0.0681) + (ExpRIPOR2 × -0.1141) + (ExpRNASET2 × 0.1453) + (ExpMAL × -0.0232) + (ExpGIMAP5 × -0.1341). The coefficients in this formula were derived from the LASSO Cox regression model. Exp[GENE] denotes the normalized expression level of the specified gene.

The TCGA-LUAD cohort was stratified into high- and low-risk groups using the median risk score as the cutoff. Kaplan-Meier analysis showed that low-risk patients had significantly better OS than high-risk patients (*P* = 0.001, Fig. 7D). External validation in the GSE68465 cohort confirmed the prognostic value of the model, with a significantly higher mortality rate in the high-risk group (*P* = 0.002, Fig. 7E). Importantly, multivariate Cox regression analysis demonstrated that the risk score was an independent prognostic factor for LUAD, independent of other clinical characteristics (Fig. S3C, D).

Dual immunofluorescence staining for BIRC3 and CD4 in lung tissues from CIP patients confirmed the co-localization of BIRC3 and CD4, verifying the presence of BIRC3⁺CD4⁺ T cells in the CIP microenvironment (Fig. 7F). Collectively, these results identify a novel CIP-CD4⁺ T-cell-based prognostic model that can effectively stratify LUAD patients for risk assessment.

### Association between CIP-CD4T-related prognostic model and tumor-infiltrating immune cells

**Fig. 8:**
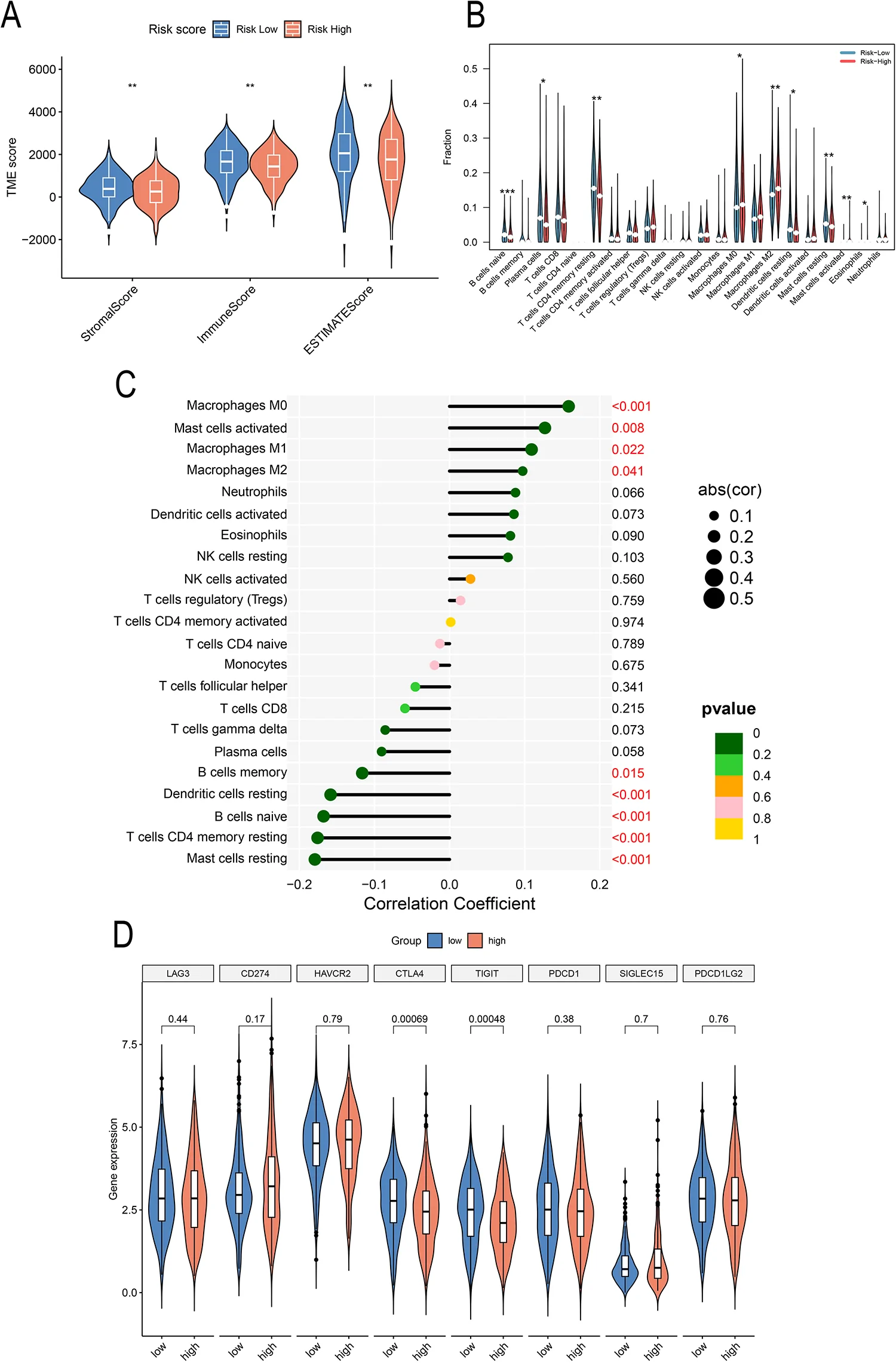
The relationship between immune cell infiltration and risk score. **A** The ESTIMATE, Stromal and IMMUNE scores in the high-risk and low-risk groups of TCGA-LUAD cohort. **B** Variations in the proportion of 22 distinct immune cell types between the high-risk and low-risk LUAD patients. **C** Correlation between the risk score and each of the 22 distinct immune cell types in LUAD patients. **D** Immune checkpoint genes expression levels of between the high-risk and low-risk groups

ESTIMATE scores showed significant differences in immune contexture between high- and low-risk groups (Fig. 8A). CIBERSORT analysis revealed that the high-risk group had higher proportions of M0 and M2 macrophages, while the low-risk group had increased mast cells, naive B cells, dendritic cells, and resting memory CD4 + T cells (Fig. 8B). Correlation analysis confirmed positive associations between risk score and M0/M1/M2 macrophages and inverse correlations with naive B cells, memory B cells, resting mast cells, and resting memory CD4 + T cells (Fig. 8C). Furthermore, immune checkpoint gene expression levels were evaluated, and the low-risk group showed significantly higher levels of TIGIT and CTLA4 than the high-risk group (Fig. 8D). These results collectively elucidated the divergent immune landscapes characterizing high-risk and low-risk LUAD patients.

## Discussion

Immunotherapy is currently a major component of lung cancer treatment and ICIs have shown significant efficacy against NSCLC (Aldarouish and Wang 2016; Lahiri et al. 2023; Qin and Li 2019). However, ICI usage may also result in immune-related adverse events, including the potentially lethal CIP (Pang et al. 2023; Tiu et al. 2022). In this study, we used scRNA-seq and hdWGCNA to identify key CD4 + T cell subsets and gene signatures associated with CIP, and explored their roles in regulating immunotherapy response and prognosis in LUAD.

The scRNA-seq data revealed a distinct immune landscape in patients with cancer and CIP. Consistent with the findings of Suresh et al. (2019), the CIP group had more CD4 + T cells than the control group did. Similarly, Bukhari et al. (2023)reported a substantial increase in CD4 + T cells, indicating their involvement in the pathological progression of CIP. Subclustering further identified Cluster 0 as a functionally important CD4 + T cell subset in CIP. The hdWGCNA identified a yellow module highly activated in Cluster 0, whose hub genes were enriched in T cell differentiation, cytokine production, and antiviral immune responses, supporting their critical roles in CIP-related immune dysregulation. Furthermore, through Cellchat analysis, we found that Cluster 0 acted as both a sender and a mediator in the IL-16 pathway. Elevated levels of IL-16 are present in the circulatory system and at sites of inflammation, infection, and cancer (Niewold et al. 2025). IL-16 is a multifunctional cytokine that acts as a chemoattractant for CD4⁺ T cells, preferentially mediating the migration of Th1 cells to sites of inflammation by binding to the CD4 receptor (Cruikshank et al. 2003; Lynch et al. 2003; Zhu et al. 2024) Studies have shown that IL-16 is involved in the intense and potentially harmful inflammatory response to MRSA pneumonia, and neutralization of IL-16 helps to improve MRSA clearance and pathology (Ahn et al. 2014). However, the mechanism of IL-16 in CIP remains unclear, and further studies are needed to clarify its specific function and its role in the occurrence and development of irAEs.

Based on these CIP-related CD4 + T cell genes, we stratified LUAD patients into two molecular subtypes. The high-expression subtype was associated with favorable prognosis and enhanced ICI response, as supported by lower TIDE scores and higher IPS scores. GSEA further revealed enrichment of immune activation pathways in this subtype, suggesting that CIP-related CD4 + T cell features may reflect a more active anti-tumor immune state. A growing body of clinical evidence supported a positive association between irAEs and improved clinical outcomes in patients receiving immune checkpoint inhibitors (Raynes et al. 2023; Zhao et al. 2021). A meta-analysis of NSCLC patients treated with anti-PD-1 therapy demonstrated that those who developed irAEs had significantly better survival and response rates compared to those who did not (Zhao et al. 2021). This finding suggests that the occurrence of irAEs may serve as a clinical indicator of robust systemic immune activation—which, although occasionally dysregulated and manifesting as autoimmunity, simultaneously reflects effective anti-tumor immune surveillance. The CIP-CD4⁺ T-cell gene signature identified in our study may capture a state of heightened immune responsiveness rather than a purely pathological autoimmune phenotype. Further studies are needed to dissect the specific molecular pathways that distinguish protective anti-tumor immunity from pathological autoimmunity, thereby providing a rationale for uncoupling therapeutic efficacy from toxicity in LUAD patients receiving immunotherapy.

We then constructed a robust 11-gene prognostic signature, with BIRC3 as the top risk gene. BIRC3 (cIAP2) is a member of the inhibitor of apoptosis protein family that regulates cell survival, immune signaling, and inflammatory responses (Frazzi 2021). In hepatocellular carcinoma, BIRC3 promotes proliferation, migration, and epithelial–mesenchymal transition via MAP3K7 and ERK signaling (Fu et al. 2019). Interestingly, few studies have focused on BIRC3 in CD4 + T cells, as revealed in our study. RNASET2 expression is also a risk factor for LUAD. RNASET2 potentially functions as a chemoattractant for macrophages and modulates the inflammatory process (Acquati et al. 2019). Previous studies have suggested that granulocytes release RNASET2, suggesting its early involvement in antimicrobial activity. As a molecular alarm, RNA-SET2 signals danger locally to the innate immune system, such as in the context of bacterial infections, thereby triggering an appropriate host response. Our findings also showed a possible correlation between RNASET2 and CD4 + T cell immunity (Baranzini et al. 2019). Gimap5 plays a key role in shaping the behavior of CD4 + T cells, affecting their activation, proliferation, and survival. Its influence on CD4 + T cells primarily stems from its ability to modulate glycogen synthase kinase-3β (GSK3β) activity after T-cell activation. The lack of Gimap5 leads to sustained activation of GSK3β, imposing restrictions on crucial processes within CD4 + T-cells (Patterson et al. 2018).

The prognostic model showed good performance in the TCGA-LUAD cohort and an external validation cohort (GSE68465), suggesting its potential therapeutic utility. Evidence suggested that the occurrence of irAEs is a positive prognostic indicator of immunotherapy outcomes, suggesting that irAEs reflect an underlying state of systemic immune activation that may also contribute to effective anti‑tumor immunity (Zhao et al. 2021). Raynes et al. showed that cutaneous, endocrine, gastrointestinal, and hepatic irAEs were associated with longer progression‑free survival and overall survival in NSCLC patients treated with pembrolizumab (Raynes et al. 2023). Furthermore, the prognostic and predictive utility of our gene signature was empirically validated in the TCGA‑LUAD cohort and an independent external validation cohort, providing robust statistical support for this extrapolation. Therefore, monitoring and early identification of irAEs may help clinicians identify patients who are more likely to benefit from immunotherapy while promptly managing toxicities. Future functional studies are needed to elucidate the specific roles of these CD4⁺ T‑cell subsets in anti‑tumor immunity and irAE pathogenesis.

The immune cells infiltrating the tumor microenvironment significantly influence immunotherapy and tumor metastasis (Galli et al. 2020; Zhang and Zhang 2020). The risk score of the prognostic model was positively correlated with the infiltration of immunosuppressive cells, whereas it was negatively correlated with immune effector cells, such as resting memory CD4 + T cells and plasma cells. Cancer immunotherapy based on ICIs mainly targets PD-1 and CTLA-4 checkpoints (Buchbinder and Desai 2016; Rotte 2019), and exhausting markers are among the other potential therapeutic targets that are currently under consideration (Bianco et al. 2019; De Giglio et al. 2021). ICIs augment T cell effector function and activation, thereby increasing the efficacy of cancer immunotherapy. Generally, tumors that express high levels of immune checkpoints respond better to ICIs, resulting in improved clinical outcomes. In our study, the low-risk group exhibited significantly higher levels of both CTLA4 and TIGIT, suggesting a more robust immunotherapeutic response. Among the risk genes, BIRC3 had the highest Hazard ratio. Baculoviral IAP repeat-containing 3 (BIRC3), also known as cIAP2, is a member of the inhibitor of the apoptosis protein (IAP) family, which plays a critical role in regulating cell death and survival. BIRC3 has garnered attention in lung cancer research because of its involvement in the inhibition of apoptosis, immune evasion, and resistance to therapy, making it a potential target for therapeutic interventions. However, previous studies on BIRC3 have primarily focused on tumor cells. Our study is the first to suggest that BIRC3 is also present in CD4 + T cells, where it plays a role in CIP and poor prognosis.

However, this study has several limitations. First, the single-cell RNA sequencing cohort included only 11 CIP patients and 6 controls, and the relatively small sample size may limit the generalizability of our findings. Larger independent cohorts are needed in the future to validate the identified CD4⁺ T cell subsets and gene signatures. Second, our evaluation of immunotherapy response mainly relied on computational algorithms such as TIDE and IPS. Although these methods can predict immunotherapy responses based on bulk transcriptomic profiles, they cannot fully capture the complexity of actual patient responses to ICIs. Future studies incorporating prospective or retrospective real-world immunotherapy cohorts with well-defined clinical outcomes are needed to more reliably validate the predictive value of the CIP-derived gene signature. Third, this study was primarily based on bioinformatics analyses. Subsequent functional experiments using in vitro or in vivo models are required to further investigate the roles of key genes such as BIRC3, RNASET2, and GIMAP5 in CD4⁺ T cell-mediated immune responses and the pathogenesis of CIP. Despite these limitations, this study provides new insights into the association between CIP and immunotherapy response.

## Conclusion

In conclusion, using single-cell transcriptomic analysis and hdWGCNA, we identified key CD4 + T cell subpopulations and a novel gene signature associated with CIP that modulate immunotherapy response and prognosis in LUAD. Our findings elucidate the immune mechanisms underlying CIP and highlight the critical role of CD4 + T cells in lung cancer immunity. This signature may serve as a promising biomarker for risk stratification and immunotherapy guidance, providing potential targets for the clinical intervention of CIP to improve patient outcomes.

## Supplementary Information

Below is the link to the electronic supplementary material.

Supplementary Material 1

Supplementary Material 2

## Data Availability

The datasets used and/or analysed during the current study are available from the corresponding author on reasonable request.
