# Supplementary material for: Single-cell hdWGCNA identifies checkpoint inhibitor pneumonitis-linked CD4 + T-cell subsets driving immunotherapy response and prognosis in lung cancer
Source: J Cancer Res Clin Oncol. 2026 Jul 10;152(9):186. doi: 10.1007/s00432-026-06522-9 (PMC13612783; doi:10.1007/s00432-026-06522-9)
Supplement: Supplementary file 1 [file 432_2026_6522_MOESM2_ESM.docx]

**Fig. S1.** scRNA-sequencing of ICIP patients. (A) Dimensionality reduction clustering analysis was used after using harmony to remove batch effects. (B) The red dots represent the identified 3000 highly variable genes. (C) The UMAP plot of scRNA-seq data. (D) The different cell types were identified by marker gene expressions.

**Fig. S2.** Hdwgcna of CD4+ T cells. (A) The top hub genes of the five gene modules. (B) Highly variable genes identified. (C) Cell trajectory map represented in pseudotime order. (D) Cell trajectory map represented in state order.

**Fig. S3. Construction and validation of ICIP-CD4T-related prognostic model. (**A) The risk score distribution plot in TCGA-LUAD cohort. (B) The risk score distribution plot in. GSE68465 cohort. (C) Univariate Cox regression for risk score and clinical factors in TCGA-LUAD cohort. (D) Multivariate Cox regression for risk score and clinical factors in TCGA-LUAD cohort.

**Fig. S4. Schematic diagram of the gene selection pipeline.**

HdWGCNA, high-dimensional weighted gene co-expression network analysis; TCGA, the Cancer Genome Atlas; LUAD, lung adenocarcinoma; LASSO, least absolute shrinkage and selection operator.
